# Ameliorative Role of Vitamin C against Cypermethrin Induced Oxidative Stress and DNA Damage in *Labeo rohita* (Hamilton, 1822) Using Single Cell Gel Electrophoresis

**DOI:** 10.3390/toxics12090664

**Published:** 2024-09-12

**Authors:** Sana Ullah, Amina Zuberi, Imdad Ullah, Mahmoud M. Azzam

**Affiliations:** 1 Fisheries and Aquaculture Lab, Department of Zoology, Faculty of Biological Sciences, Quaid-i-Azam University, Islamabad 45320, Pakistan; 2 Department of Zoology, Division of Science and Technology, University of Education, Lahore 54000, Pakistan; 3 Department of Biosciences, Durham University, D86, Durham DH1 3LB, UK; imdad.ullah@durham.ac.uk; 4 Durham Genome Center, Lanchester DH7 0EX, UK; 5 Department of Animal Production, College of Food and Agriculture Sciences, King Saud University, Riyadh 11451, Saudi Arabia; mazzam@ksu.edu.sa

**Keywords:** cypermethrin, rohu, reactive oxygen species, lipid peroxidation, DNA damage, single cell gel electrophoresis

## Abstract

The present study was undertaken to evaluate cypermethrin (CYP)-induced oxidative stress [reactive oxygen species (ROS) and lipid peroxidation (LPO) in gills, muscles, brain, and liver tissues] and DNA damage/genotoxicity (peripheral blood erythrocytes) in a freshwater teleost rohu (*Labeo rohita*) and the protective role of vitamin C. The LC_50_ of CYP against rohu was found to be 4.5 µg/L in a semi-static culture system through probit analysis. Fingerlings of rohu were distributed into four groups (Group 1st served as a control, fed 35% protein basal diet and was not exposed to CYP; Group 2nd was fed a basal diet and exposed to CYP; Group 3rd and Group 4th were fed diets supplemented with vitamin C at the rate of 100 and 200 mg/kg diet, respectively, and exposed to CYP). Fingerlings were reared on a basal and vitamin C-supplemented diet for 28 days prior to exposure to CYP. The results indicate a time-dependent significant increase in ROS and LPO (indicated by time course increase in TBARS level) as well as DNA damage in terms of number of comets, % DNA in tail, tail moment, tail length, and olive tail moment after exposure to LC_50_ of CYP. However, statistically comparable results in both Groups 1st and 4th indicate the protective role of vitamin C. The results reveal the effectiveness of vitamin C as a feed additive for countering pesticides toxicity in *Labeo rohita*. The current study indicates CYP as a potential genotoxicant for fish and classifies SCGE as a reliable and sensitive tool for assessing DNA damage.

## 1. Introduction

Pesticides are used in agricultural fields against pests in order to increase the production of different crops, although these are very toxic for other classes of animals [1]. Pesticides are a major cause of air pollution. They also contaminate aquatic ecosystems, posing significant risks to non-target aquatic organisms including fish. Pesticides and their residues infiltrate the food chain, and subsequently reduce biodiversity and disrupt ecological balance [2]. These contaminants, as well as their modes of action, leaching mechanisms and toxic effects, are well documented, and are considered as a major issue of concern from the local to the global scale [3]. With the emergence of resistance in pests, different classes and groups of pesticides are employed, with a continuous increase in their number. Recently, there has been a notable surge in the commercialization and use of synthetic pyrethroids (SPs) because of their efficacy, and we have seen a reduction in the use of more hazardous carbamates, as well as organochlorine and organophosphate insecticides [4,5]. Pyrethroids are esters of chrysanthemic acid and are reported in almost all spheres of ecosystem on account of their widespread use in farms, agriculture, nurseries, gardens, homes, commercial products (such as pet shampoos), and public and animal health programs [6]. Over a thousand pyrethroids are currently in use across the globe [7]. Pyrethroids account for about 20% of the global insecticide market and their global sale value is estimated to be USD 1.3 billion [8].

Cypermethrin (CYP) is one of the most extensively employed and highly effective synthetic cyanophenoxybenzyl pyrethroids [9]. CYP is a synthetic insecticide derived from the natural compound pyrethrin, which itself is isolated from Chrysanthemum flowers, *Chrysanthemum cinerariaefolium* and *Chrysanthemum coccineum* [10]. To enhance their stability, efficacy, and duration of action, synthetic derivatives of pyrethrin were developed, including CYP. CYP is commonly used in all types of agriculture, buildings, forestry, gardens and farmyards to prevent, deter, repel, or kill insects [11]. It is commercially used to prevent cotton pests and soyabean pests [12]. Its use for repelling and controlling mosquitos has proven to be very effective in preventing malarial parasites [13]. But tragically, CYP is misused, and illegally employed in different parts of the world for fishing purposes, including in Pakistan [14]. It is also utilized as a chemotherapeutic agent by aquatculturists/fish farmers to control parasitic infections of copepods [15]. Therefore, this large-scale application of CYP is resulting in major threats to water quality and aquatic ecology, and residing in commercially valuable aquatic organisms, such as in fish.

Studies have reported different ranges of the concentration of CYP in different aquatic bodies, such as 0.71 to 1.97 ng/g (dry weight) in sediments [16], 2.1–4.4 ng/L in seawater [17], 8.115 to 15.46 mg/L in surface water, 4.48 to 12.18 mg/L in groundwater [18], up to 492 ng/L in freshwater during storm events [19], and up to 29.43 mg/L in surface water in a lychee-producing area [20]. Similarly, different reports revealed different amounts of CYP being sold across the globe, such as in the UK, 24,000 kg of CYP was used for agricultural purposes, whereas 13,000 kg was sold for veterinary medical purposes in 2016 [21]. A study from Pakistan revealed the presence of CYP in the soil of both rainfed and irrigated areas, ranging from 0.14 to 27.62 mg/kg and from 0.05 to 73.75 mg/kg, respectively [22]. A recent research study reported CYP in potable water from five different districts of Punjab, with a maximum concentration of 1589 ng/L [23].

Fish are very sensitive to changes in environment; therefore, they are considered as the best sentries for evaluating the impacts of contaminants and associated risks in aquatic environment [24]. Currently, different fish species (such as zebrafish, medaka, fathead minnow, and rohu) are widely employed as models in ecotoxicology, aquatic toxicology, and the risk assessment of emerging or newly introduced chemicals, pollutants, or toxicants. Of these contaminants, pesticides are of major interest for environmental scientists on account of their stability, potential to contaminate aquatic environments even at sub-lethal concentrations, and easy bio-accumulation in the tissues of fish [25].

The mutagenic and genotoxic impacts of pesticides on non-target organisms including fish, their rapid expansion, and their influence on the environment are matters of grave concern around the globe [26]. Genotoxicants become more hazardous when they have bio-accumulative properties and enter into food chains [27]. Different species of fish are used as suitable models for evaluating the genotoxic impacts of pollutants [28]. The eco-toxicological characteristics of rohu (*Labeo rohita*; Hamilton, 1822), including easy availability, easy maintenance in a wet lab, commercial importance, and wide distribution, make it a brilliant aquatic model for toxicological research. Research on genotoxicity revealed that genotoxic effects significantly impact fitness traits, genetic patterns, population dynamics, and reproductive success [29]. Subsequently, the occurrence of genotoxic chemicals in aquatic bodies is a growing concern around the globe, which necessitates the development of methods to detect genotoxicity induced by these chemicals in aquatic organisms as soon as possible [30].

Comet assay or single-cell gel electrophoresis (SCGE) can detect alkali labile sites and DNA strand breaks (both double and single) through quantifying DNA migration from nuclear immobilized DNA [31]. SCGE is considered an important, sensitive, rapid and reliable technique for the bio-monitoring of environmental contaminants and measuring DNA damage in any tissues irrespective of their mitotic activity [27,32]. Previous studies identified the comet assay as the best assay for detecting genotoxic effects in the field, as well as in a laboratory [25]. SCGE is a widely employed assay for use in aquatic organisms, both invertebrates and vertebrates, when detecting chromosomal or genetic damage induced by exposure to genotoxic chemicals [33]. 

Information regarding the DNA-damaging capability of CYP in fish is scant, specifically as regards data concerning the acute genotoxic effects on rohu. Therefore, the present study was carried out to evaluate CYP-induced DNA damage in peripheral blood erythrocytes of *L. rohita* using SCGE/comet assay. DNA damage is often associated with oxidative stress; therefore, key parameters of oxidative stress, including the production of reactive oxygen species and the induction of lipid peroxidation, were also appraised. Moreover, the current study evaluated the recuperative role of vitamin C (a vital antioxidant) against CYP-induced oxidative stress and DNA damage to find out if its inclusion in fish feed can mitigate the toxic effects. Based on the novelty and significance of the current study, this article will be a valuable resource for researchers, environmental practitioners, and policymakers seeking to address the complex issues surrounding pesticides-induced genotoxicity/DNA and the mitigation of these issues using a commonly available antioxidant (Vitamin C). 

## 2. Materials and Methods

### 2.1. Transport and Acclimatization of Labeo rohita

A total of 250 uniform-sized, active and healthy fingerlings of rohu, *Labeo rohita* (average body weight 6.55 ± 1.01 g and average length 8.17 ± 0.794 cm), were collected from Faisalabad Fish Hatchery, and were transported to Fisheries Research Station, Quaid-i-Azam University by adopting the closed-system, live hauling method. Prior to shifting fish to circular tanks (fiberglass) from oxygenated bags, tempering was carried out by steady and regular water mixing from circular tanks in polyethylene bags. To avoid any damage, on account of jumping due to journey stress, special care was taken while handling the fish. The tanks containing dechlorinated and well-oxygenated water were given protection with nets. Then, after two days, the fish were shifted to aquaria (60 × 30 × 30 cm), previously washed with table salt in order to free the walls of the growth of microbes. The fish were acclimatized for 15 days before starting the experiment. 

During the acclimatizing of fish, they were fed to satiation with 35% protein-based small pelleted feed (Table S1), twice a day (5% of their body). The undigested and unutilized feed and excreta were siphoned off daily to avoid stress and stressful conditions by ensuring optimum water quality. The water quality factors, such as temperature, dissolved oxygen, salinity, pH, electrical conductivity, etc., were assessed on a regular basis to ensure their optimum ranges. Dead specimens were removed from the aquaria as soon as they were observed so as to avoid water quality deterioration. 

### 2.2. Experimental Toxicant—Cypermethrin

Cypermethrin [CYP; Cyano(3-phenoxyphenyl)methyl3-(2,2-dichloroethenyl)-2,2-dimethyl-cyclopropane-carboxylate] from Sigma-Aldrich purchased from AMS Traders (Islamabad Pakistan) was used for preparing the stock solution. The CYP stock solution was prepared by dissolving 5 mg of CYP in 5 mL of acetone (80%), which is non-toxic to fish, as fish do not take it up from the surrounding water [26]. Then, 1 mL of it was taken in a 10 mL volumetric flask with the help of a micropipette, and water was added up to mark to make a concentration of 1 mg CYP per 10 mL. A further dilution was performed using the stock solution. The concentration 4.5 µg/L was identified as the median lethal concentration (LC_50_), causing 50% mortality for 96 h.

### 2.3. Experimental Design

The experiment was carried out in a semi-static closed system. Healthy, active and uniform-sized fish irrespective of sex were chosen and equally distributed in twelve glass aquaria (60 × 30 × 30 cm) at a stocking density of 1.5 kg/m^3^ after acclimatization. The experiment was carried out in replicates of three, comprising four groups.

First group: Fish fed a 35% protein diet (Table S1) and not exposed to pesticide.
Second group: Fish fed a 35% protein diet and exposed to CYP.
Third group: Fish fed a 35% protein diet supplemented with vitamin C (100 mg/kg diet) and exposed to CYP.
Fourth group: Fish fed a 35% protein diet supplemented with vitamin C (200 mg/kg diet) and exposed to CYP.

After 28 days of feeding of basal and vitamin C-supplemented diets to respective groups, the fish in the three groups except the control were exposed to an acute concentration, 4.5 µg/L of CYP, while the first group (control group) received 0.005% acetone, equal to its volume used in treatment groups. The water in all the aquaria was changed after every 24 h and the CYP concentration was restored afresh. As we employed acute concentrations of CYP, the experiment was undertaken for 4 days (96 h). After every 24 h, such as at 24, 48, 72 and 96 h, 3 fish of each aquarium were captured before the exchange of water (n = 3; N = 9). These fish were stunned to death after anesthetizing them using MS222. By using sterilized instruments, their target tissues, including brain, gill, liver, and muscles tissues, were dissected out. They were kept in Ziploc bags and frozen in liquid nitrogen before assaying their ROS. The dissected tissues were stored at −20 °C for lipid peroxidation assaying. Blood was collected through caudal vein puncture using a sterile needle [25 G—0.5 mm (needle diameter or thickness) × ½ inch or 13 mm (needle length)] and syringe. The collected blood was shifted to EDTA (anticoagulant) tubes. The blood samples were stored at 4 °C until processing. The DNA damage was assessed through comet assay. 

### 2.4. Reactive Oxygen Species (ROS)

ROS generation was appraised via DCF-DA (2′,7′-dichlorofluorescein diacetate) by following Ullah et al. [34]. The extracted tissues (gills, muscles, brain, and liver) were pooled (0.1–1 g wet weight) because of their smaller size (separately; for example, liver from the same group was pooled together to attain the required weight of the tissues; n = 3 from each aquarium; N = 9), followed by their incubation in DCF-DA (10 µM; 100 mL) in methanol in a water bath (30 min, 37 °C). The DCF fluorescence was observed through a spectrophotometer [525 nm (emission) and 488 nm (excitation) wavelengths]. The standard curve used for the fluorescence value was 0.500 nM DCF. 

### 2.5. Lipid Peroxidation Assay (LPO/TBARS)

The LPO activity was assayed by following the method of Wright et al. [35] in the gills, liver, brain, and muscle tissues in all the groups (24 through 96 h). The LPO assay typically uses thiobarbituric acid (TBA) to react with malondialdehyde (MDA), a product of lipid peroxidation. The TBA-MDA adduct absorbs light at a maximum of 535 nm. To prepare a reaction solution for LPO, supernatant is required, which is the clear liquid layer that remains after centrifugation (spun at high speed) of gills, liver, brain, and muscle tissues. The heavier particles, including cell debris, nuclei, and mitochondria, are separated and form a pellet at the bottom of the tube after centrifugation, whereas the clearer liquid portion above the pellet is called as supernatant. However, before centrifugation, the tissues were homogenized in lysis buffer (phosphate buffer saline) for the accurate measurement of MDA. In the LPO assay, two measurements are typically required to measure the change. The first measurement was performed immediately after sample preparation (e.g., homogenization; within 10–30 min of sample preparation). The initial measurement serves as a baseline or control value, which accounts for any pre-existing lipid peroxides and is used to normalize the final measurement. The second measurement was performed after incubation with pro-oxidant (i.e., ascorbic acid). This final measurement reflects the change in lipid peroxidation levels due to the reaction. To prepare the reaction mixture of 1.0 mL, phosphate buffer (pH 7.4, 0.1 M), supernatant (0.2 mL), ascorbic acid (100 mM; 0.2 mL) and ferric chloride (100 mM; 0.02 mL) were mixed, followed by incubation in a water bath (37 °C; 1 h). Trichloroacetic acid (10%; 1.0 mL) was added to stop the reaction, followed by the addition of thiobarbituric acid (1.0 mL) and the boiling of the tubes in a water bath (20 min). Then the tubes were cooled (in an ice bath) and centrifuged (10 min; 2500× *g*). Then a change in absorbance of the reaction solution was recorded at 535 nm after 1 min using a spectrophotometer to quantify the amount of MDA formed, which is directly proportional to the level of lipid peroxidation. The result was expressed as nM TBARS/min/mg tissue (37 °C) using molar extinction coefficient (1.56 × 10^5^/M cm).

### 2.6. DNA Damage

DNA damage was evaluated in blood (peripheral blood erythrocytes) by employing the neutral single-cell gel electrophoresis (SCGE)/comet assay by following Singh et al. [36] with a slight modification, as reviewed by Lee and Steinert [37]. SCGE is a simple practice used for noticing single- as well as double-strand breaks and alkali-labile sites in DNA.

#### 2.6.1. Single Cell Gel Electrophoresis (SCGE)/Comet Assay

SCGE assesses single cells embedding in agarose, cell lysis, and DNA liberation by electrophoresis that leads to the cleaving/slicing of intact DNA heads to form a comet tail. Both intact as well damaged DNA is visualized through a fluorescent microscope, followed by their scoring through image-analyzing software. Briefly, for carrying out the comet assay, microscopic slides (layered with cells in agarose) were prepared, and membrane lysis (for DNA release), the formation of single-stranded DNA (alkali contact, pH 13), electrophoresis (neutral), the staining of DNA, analysis, and the scoring of the comets were performed. To avoid the induction of DNA damage, the whole process was undertaken in low light. 

#### 2.6.2. Slide Preparation

Frosted slides were moderately heated (using slide warmer) while being sheltered with RMPA (100 µL; 1% regular melting point agarose) prepared in distilled water (40 °C). The slides were straightway covered with a coverslip (20 × 50 mm). The slides were kept in a chilled tray (4 °C; 30 min) to solidify the agarose. Then the coverslips were removed and a second layer was spread over the first layer at 37 °C. The second layer was of 85 µL having 65 µL of 1% low-melting-point agarose (LMPA) and 20 µL of blood suspension (used to dilute the mixture of fish erythrocytes (red blood cells) in a buffer solution, such as phosphate buffer saline, to prevent cell agglutination, maintain cell viability, and facilitate cell separation). Then the slide was covered with a coverslip again. Then the double-layered slides were solidified for the next step of lysis of the cells.

#### 2.6.3. Lysis

During the lysis of cells (fish erythrocyte), the cells are lysed (broken open) using a lysis buffer, which typically contains detergents (such as Triton X-100) and salt (such as NaCl and EDTA) to disrupt cell membranes. Lysis refers to the process of breaking down cells to release their DNA, allowing the assessment of DNA damage. The lysis buffer dissolves cell membranes, the nuclear membrane and proteins, releasing DNA from the cell. Effective lysis is necessary for releasing intact DNA, preventing DNA degradation during the process, and ensuring the accurate assessment of DNA damage. The optimal lysis conditions vary depending on cell type; however, for fish erythrocyte, the requirements are a neutral to slightly alkaline pH, a temperature of 4 to 37 °C, and a time duration of 30 min to several hours.

A lysis buffer was synthesized by mixing NaCl (2.5 M), EDTA (100 mM; dissolved via NaOH pellets—0.2 g/10 mL solution), Tris Base (10 mM; pH 10.3), Triton X-100 (Dithiothreitol; 1% *w*/*v*; added just before lysis commencement). The coverslips were again removed from the prepared slides to lyse the cells by immersing the slides in a fresh cold lysis buffer in a histology jar (pH 10.3) and incubating the slides with lysing solution (24 h, room temperature), followed by sweeping the slides with distilled water (three times at 20 min intervals to remove traces of salt and detergent). Aluminum foil was used to top the jar and minimize light. 

#### 2.6.4. Neutral Electrophoresis

The prepared slides, after lysis, were placed steadily and carefully in electrophoresis tray columns (front towards anode) containing distilled water (1200 mL) and neutral electrophoresis buffer [300 mL; EDTA (0.5 M) + boric acid (27.5 g/L; dissolved via stirring at 45 °C) + Tris base (54 g/L; pH 8)], followed by their equilibration with electrophoresis buffer (20 min). Electrophoresis was carried out at 25 V (0.714 V/cm) for 20 min, followed by draining the buffer from the tank and the removal of the tray, then covering the slides (aluminum foil) and air-drying them (at 5 °C) overnight. 

#### 2.6.5. Analysis and Scoring of the Slides

The slides were rehydrated with distilled water for 60 min, followed by staining with Acridine orange (300–400 μL of 20 μg/mL of distilled water). After staining the slides, they were examined under an epifluorescent microscope (400X, Nikon AFX-1 Optiphot; Attached with the microscope—Tokyo, Japan made). Digital images were taken for succeeding analyses/scoring with TRITEK CometScore 1.5. software. For analysis, 120 cells were counted from four fields of each slide, counting intact DNA and the numbers of comets. DNA comet parameters including comet length (CL, μm), head length (HD, μm), % DNA in head (%H), tail length (TL, μm), tail DNA (TDNA, %), tail moment (TM, μm) and olive moment (OTM, μm) were recorded for assessing DNA damage. 

### 2.7. Statistical Analysis

The results, expressed as mean ± S.E., were analyzed through one-way analysis of variance (ANOVA), followed by HSD Tukey test for testing the homogeneity of variance (multiple variance analysis) in Statistix (V. 8.1). *p* values of less than 0.05 were considered significant statistically. 

## 3. Results

### 3.1. Reactive Oxygen Species

**Figure 1:**
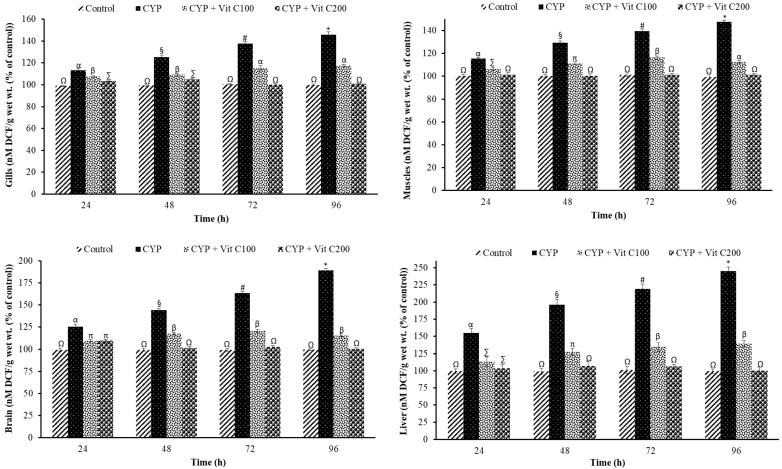
Reactive oxygen species (ROS) in gills, muscles, brain, and liver tissues of rohu, *Labeo rohita* (nM DCF/g wet wt.; % of control). Data (presented as mean ± S.E.; N = 9) were analyzed using ANOVA followed by HSK Tukey test. Different symbols on the bars show significant difference at *p* < 0.05, whereas the same symbol indicates no significant difference.

A time-reliant significant increase in ROS was observed in the gills, brain, muscles, and liver of rohu in the second group (fed with basal diet and exposed to CYP), as shown in Figure 1. However, statistically comparable results were observed in the first group (control, not exposed to CYP) and the fourth group ([fed vitamin C-enriched diet (200 mg/Kg diet)]; however, ROS production was restricted in the third group as compared to the second group. 

### 3.2. Lipid Peroxidation Assay (LPO/TBARS)

**Figure 2:**
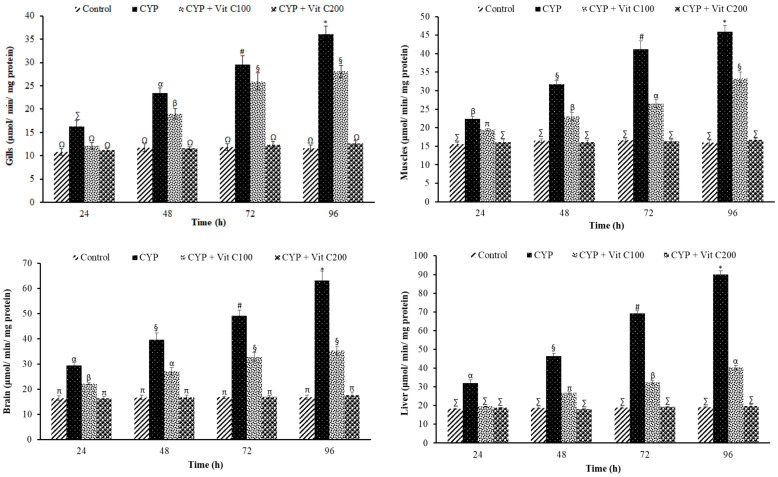
Lipid peroxidation (LPO; μmol/min/mg protein) levels in gills, brain, muscles, and liver tissues of rohu, *Labeo rohita*. Data (presented as mean ± S.E.; N = 9) were analyzed using ANOVA followed by HSK Tukey test. Different symbols on the bars show significant difference at *p* < 0.05, whereas the same symbol indicates no significant difference.

The levels of LPO observed in various tissues of the control and experimental groups at various time intervals after CYP exposure are presented in Figure 2. In gills, muscles, brain, and liver tissues, CYP exposure caused the highest time-dependent increase in LPO level in a group of fish fed the basal diet (Group 2nd), followed by fish fed a diet supplemented with vitamin C at the rate of 100 mg/Kg diet (Group 3rd). However, the LPO levels in different tissues in Group 1st (control, not exposed to CYP) and Group 4th fed a vitamin C enriched diet (200 mg/Kg diet) were similar, and higher than those observed in Group 2nd and Group 3rd.

### 3.3. DNA Damage

**Figure 3:**
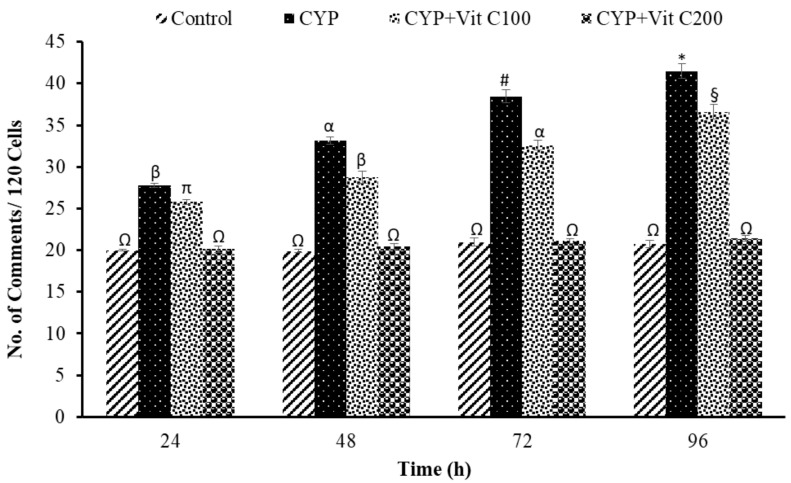
Number of comets per 120 cells observed in peripheral erythrocytes of rohu, *Labeo rohita*. Data (presented as mean ± S.E.; N = 9) were analyzed using ANOVA followed by HSK Tukey test. Different symbols on the bars show significant difference at *p* < 0.05, whereas the same symbol indicates no significant difference.

**Figure 4:**
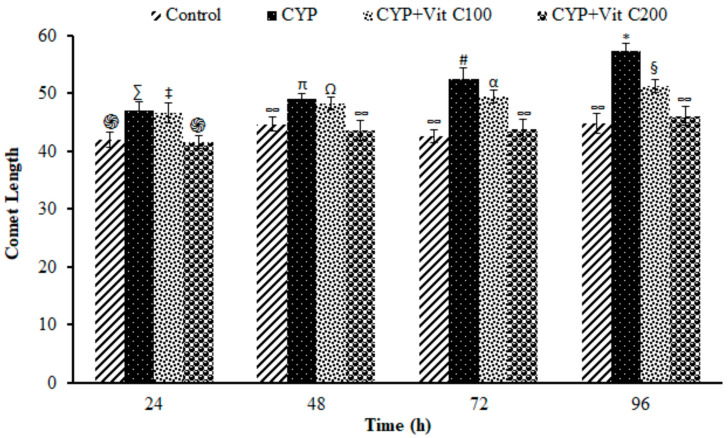
Length (µm) of comets formed in peripheral blood erythrocytes of rohu, *Labeo rohita*. Data (presented as mean ± S.E.; N = 9) were analyzed using ANOVA followed by HSK Tukey test. Different symbols on the bars show significant differences at *p* < 0.05, whereas the same symbol indicates no significant difference.

**Figure 5:**
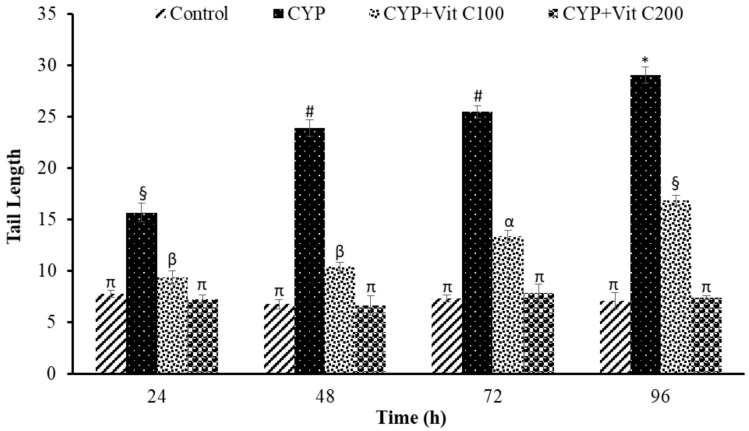
Tail length (µm), formed in peripheral erythrocytes of rohu, *Labeo rohita*. Data (presented as mean ± S.E.; N = 9) were analyzed using ANOVA followed by HSK Tukey test. Different symbols on the bars show significant differences at *p* < 0.05, whereas the same symbol indicates no significant difference.

**Figure 6:**
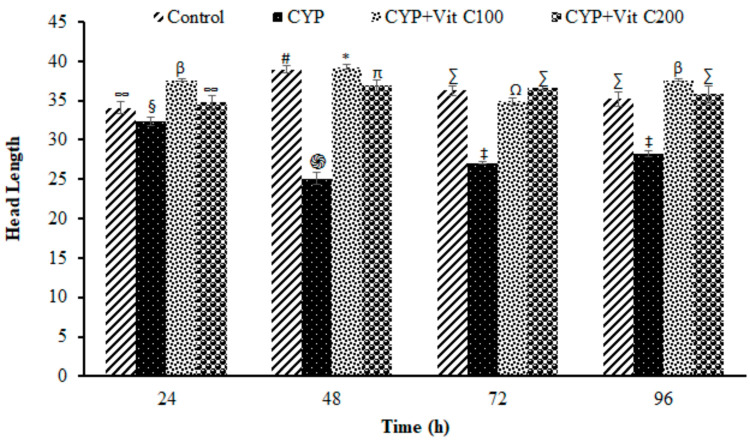
Head length (µm) of comets in peripheral erythrocytes of rohu, *Labeo rohita*. Data (presented as mean ± S.E.; N = 9) were analyzed using ANOVA followed by HSK Tukey test. Different symbols on the bars show significant differences at *p* < 0.05, whereas the same symbol indicates no significant difference.

**Figure 7:**
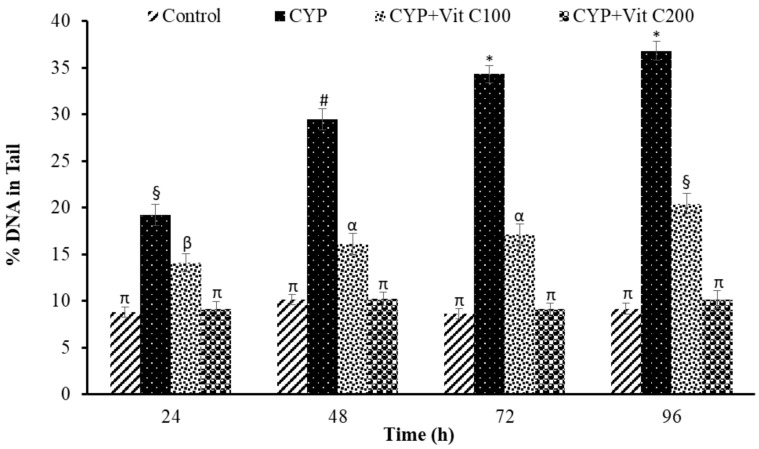
Percent DNA in tail (%) of comets in peripheral erythrocytes of rohu, *Labeo rohita*. Data (presented as mean ± S.E.; N = 9) were analyzed using ANOVA followed by HSK Tukey test. Different symbols on the bars show significant differences at *p* < 0.05, whereas the same symbol indicates no significant difference.

**Figure 8:**
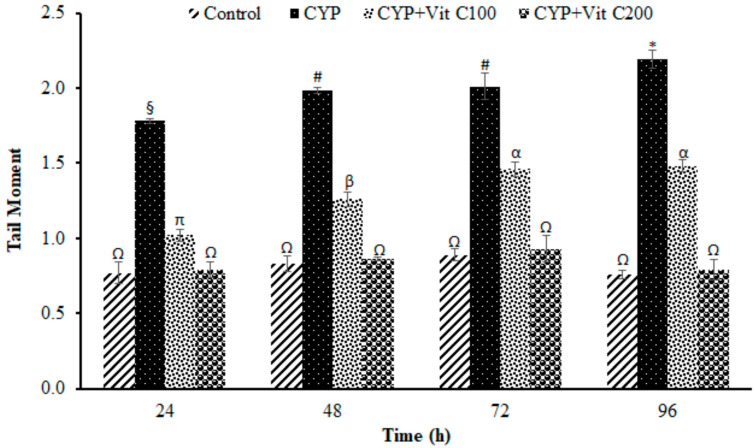
Tail moment of comets in peripheral erythrocytes of rohu, *Labeo rohita*. Data (presented as mean ± S.E.; N = 9) were analyzed using ANOVA followed by HSK Tukey test. Different symbols on the bars show significant difference at *p* < 0.05, whereas the same symbol indicates no significant difference.

**Figure 9:**
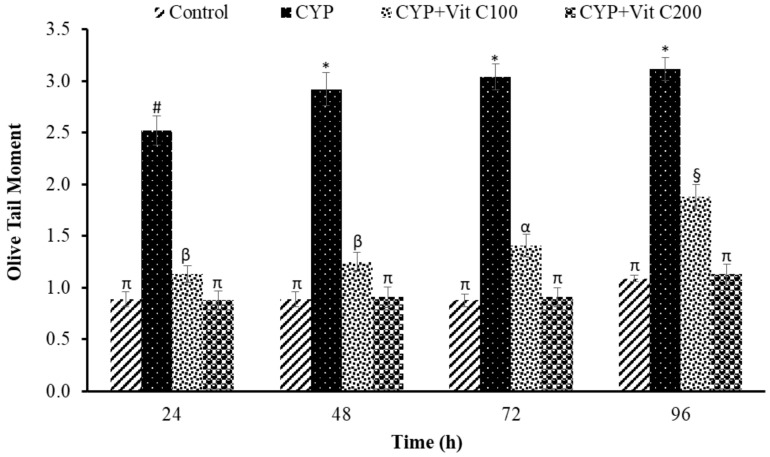
Olive tail moment of comets in peripheral erythrocytes of rohu, *Labeo rohita*. Data (presented as mean ± S.E.; N = 9) were analyzed using ANOVA followed by HSK Tukey test. Different symbols on the bars show significant difference at *p* < 0.05, whereas the same symbol indicates no significant difference.

DNA damage in the peripheral blood erythrocyte of rohu was observed to be time-dependent. DNA damage was assessed via different parameters, including observed number of comets/120 cells, comet lengths (µm), head length (µm), tail length (µm), head DNA (%), tail DNA (%), tail moment and olive tail moment in all groups, as shown in Figure 3, Figure 4, Figure 5, Figure 6, Figure 7, Figure 8 and Figure 9. Figure S1 shows the percent of DNA in the head, whereas Figure S2 shows DNA damage observed in Group 2nd and Group 3rd and intact DNA in Group 1st and Group 4th.

## 4. Discussion

The LC_50_ of CYP against rohu for 96 h was observed to be 4.5 µg/L in the current study, which is in the same range as that derived in an earlier study reporting 3.48 µg/L as the LC_50_ of CYP against rohu fingerlings [38]. Changes in LC_50_ values for different fish species or even for the same species might be due the pesticides’ formulation, as well as the stereochemistry of their molecules. A pesticide using a single isomer base is relatively more toxic as compared to one using various isomers combinations in their formulation. Further, this pesticide’s toxicity is also correlated with its content of inert ingredients, contaminants and active ingredients. Moreover, the toxicity of a chemical to fish is also dependent on various other factors, such as the size, age and health of the fish, and prevailing temperature. According to Singh et al. [39], pyrethroids are more toxic in winter as compared to summer, and approximately tenfold differences were observed in LC_50_ values at 96 h at 10, 15 and 20 °C.

### 4.1. Reactive Oxygen Species

Synthetic pyrethroids (SPs) induce oxidative stress via the production of ROS [34]. Increased ROS levels are associated with different biochemical changes, including the modification of proteins, antioxidants, lipids, and nucleic acids such as DNA [40]. Increases in reactive oxygen species lead to oxidative stress, which subsequently leads to cellular and physiological damage. Reactive oxygen species interrupt cellular membranes and make them leaky, which subsequently leads to various physiological instabilities followed by programmed or uncontrolled cell death [41]. The current study also revealed a significant and time-dependent increase in ROS after exposure to CYP in different tissues of rohu. The increase was in correspondence with a time-reliant increase in lipid peroxidation, as reported by previous studies [42]. Similarly, a significant increase in the DNA damage was in congruence with the increase in ROS, as reported by previous studies [34]. The highest ROS was observed in the liver on account of its detoxification role, followed by the brain as it has an oxidizable substrate [43]. 

ROS production in the current study might be increased after mitochondrial dysfunction (incomplete reduction of oxygen during ATP production followed by electrons leakage, reacting with oxygen to form ROS), enzyme interference (CYP might inhibit ROS-neutralizing antioxidant enzymes, including superoxide dismutase, catalase, and glutathione peroxidase, which result in ROS accumulation), or inflammatory response (the activation of immune cells such as macrophages and neutrophils produces ROS as the body’s defense mechanism; however, their excessive production leads to oxidative stress) in rohu after exposure to CYP [26]. Previous studies also revealed that synthetic pyrethroid exposure led to increased ROS production in different fish species, such as *Hypophthalmichthys molitrix* after exposure to deltamethrin [34], and *Ctenopharyngodon idella* after exposure to bifenthrin [1]. 

### 4.2. Lipid Peroxidation

The inability of an animal’s defense system to completely neutralize reactive oxygen species leads to the oxidative damage of membrane lipids by ROS [44]. Therefore, LPO is considered as a key outcome of oxidative stress, and is often employed as a key parameter in eco- or aquatic toxicology. A time-dependent increase in lipid peroxidation (LPO) was observed in different tissues of rohu after exposure to LC_50_ of CYP, indicated by a time course-dependent increase in TBARS level. This study is in congruence with preceding studies reporting increased LPO in fish after exposure to various SPs, such as *Cyprinus carpio* after exposure to γ-cyhalothrin [45], and *Clarias gariepinus* after exposure to CYP [46]. 

LPO is a multifaceted process. It displays a higher activity in polyunsaturated fatty acid-rich membranes. It causes oxidative damage to specific tissues by destroying its membranous structure by decomposing the double bonds present in unsaturated fatty acids [46,47]. The LPO level in rohu after exposure to CYP showed time-dependent and tissue-specific patterns, as LPO induction was higher in the liver, followed by the brain. The highest lipid peroxidation activity in the liver might be due to the several redox cycles and excessive ROS production in the liver. The results of the current research are in accordance with previous research reporting that CYP induced higher LPO levels in the livers of *Clarias gariepinus* [46] and *Tor putitora* [48], and higher deltamethrin in the liver of *Channa punctatus* [49] and *Hypophthalmichthys molitrix* [34]. 

### 4.3. DNA Damage Induction

The current study revealed the induction of damage in blood erythrocytes after exposure to the LC_50_ of CYP. The observed DNA damage parameters were the number of comets per 120 cells, comet length, tail length and head length, % DNA in head, % in tail, tail moment and olive tail moment. The current study clearly demonstrates the genotoxic effects of CYP on rohu by inducing a significant increase in comets’ numbers, tail lengths, % DNA in tail, and olive tail moment in the group fed with a basal diet (Group 2nd), followed by the group fed a basal diet supplement with vitamin C at the rate of 100 mg/Kg diet. No significant differences were observed in fish in Group 1st (Control) or fish in Group 4th (fed a vitamin C-enriched diet at the rate of 200 mg/Kg diet), suggesting the robust antioxidant potential of vitamin C against ROS, which is mainly responsible for disturbing the biochemical parameters of fish [50]. DNA is one of the most vulnerable among these biomolecules, as it undergoes severe damage in a shorter time period.

The DNA damage was observed to linearly increase in Group 2nd with time; however, there were no significant differences in vitamin C-fed groups (Group 4th; 200 mg/kg diet) as compared to the control group for the studied parameters. The detection of DNA damage through SCGE is based on the principle that fragmented or damaged DNA migrates more rapidly towards the anode compared to intact, undamaged DNA. This results in the formation of a tail, and gives it the shape of a comet. During electrophoresis, the DNA moves freely, and its tranquil loops are dragged out of the head or nucleus of the cell, subsequently leading to comet formation [51]. The migration of the DNA out of the head/nucleus is shown by the tail length, hence the longer the tail length, the greater the DNA damage [52]. Similarly, the smaller the DNA fragment, the further the migration. Therefore, the tail length is associated with the sizes of DNA fragments produced during the unwinding of the comet assay [53]. The percent of DNA in the tail is employed as a suitable parameter for evaluating DNA damage, as it reveals DNA that has moved out of the nucleus in a percentage [54]. Olive tail moment is employed to measure the distance of the tail from the center of the head, and is used to detect the degree of DNA damage [36].

In vertebrates, including fish, DNA damage involves the conversion of double-stranded DNA to single-strand or chromosomal aberrations, and it can be measured using the comet assay [55]. The direct relationship between ROS (such as O_2_^−^, H_2_O_2_, HOCl, O_3_, etc.), ROS-generating enzymes and DNA damage is well-established. H_2_O_2_ can cross membranes easily, thus penetrating the nucleus, and it reacts with the iron or copper ions present in the chromosome, promoting the H_2_O_2_-dependent damage of isolated DNA and DNA within chromatin by forming OH, which is highly reactive and unable to diffuse significant distances within cells. Paravani et al. [56] also observed H_2_O_2_ to be the major DNA-damaging reactive oxygen species in the gill cells of zebrafish. DNA damage might be linked with the metal ions released within the cell as a result of oxidative stress, which bind to the DNA [26]. Thus, just as oxidative stress elevates intracellular free Ca^2+^, it might increase the numbers of intracellular free iron and/or copper ions that could bind to DNA and make it a target for oxidative damage.

The genotoxicity observed in the current study confirms the findings of previously conducted research on DNA damage induction in different fish species after exposure to various toxicants, such as *Clarias gariepinus* [57], *Carrasius auratus* [58], *Hyphssobrycon luetkenii* [59], *Oreochromis mossambicus* [60], *Channa punctatus* [61], *Labeo rohita*, *Ctenopharyngodon idella*, *Catla catla* and *Cirrhina mrigala* [62], and *Danio rerio* [56].

### 4.4. Protective Role of Vitamin C

It has been effectively established in various previous studies that DNA damage is directly associated with ROS and elevated levels of LPO [1,34]. Vitamin C is a powerful antioxidant (water soluble) and performs a variety of non-enzymatic actions. It reduces harmful oxidants, including ROS (i.e., H_2_O_2_), and hence protects low-density lipoproteins from oxidation [41,63]. Due to the greater integrity of the lipoprotein, H_2_O_2_ cannot easily penetrate biological membranes, thus it cannot approach the nucleus and consequently cannot react with free copper or iron ions. This inhibits OH formation as well, and protects DNA from the most potent causes of DNA damage via the Fenton reaction [64,65]. The effective role of vitamin C against ROS degradation, as a protector and as a strong antioxidant, is well established [41]. Moreover, a plethora of the literature supports the protective, recuperative, or ameliorative roles of vitamin C against DNA damage in terms of chromosomal aberration, damage induced in micronuclei, and DNA strand breakage [65,66]. Thus, the current study revealed that vitamin C inhibits the production of ROS after exposure to CYP, and subsequently prevents lipid peroxidation in gills, muscles, brain, and liver tissues, as indicated in previous studies [67,68]. Similarly, because of the inhibition of ROS production, the DNA was protected, and hence no DNA damage was observed in Group 4th, fed with a vitamin C-rich feed (200 mg/Kg). Vitamin C might protect the DNA by donating its electrons or via its anti-clastogenic effect [69]. Ghazanfar et al. [70] reported the protective effects of vitamin C against fipronil- and buprofezin-induced genotoxic damage in *Cyprinus carpio*. Previous studies on vitamin C also revealed its protective effects against cypermethrin-induced hematological and biochemical toxicities in *Heteropneustes fossilis* [9].

## 5. Conclusions

The current study clearly classifies CYP as a potent genotoxicant and mutagen. The issue of most grave concern is that the LC_50_ observed in the current study falls short of some of the reported real-world concentrations of CYP in surface water across different parts of the world. CYP was observed to be highly toxic to rohu at this concentration (4.5 µg/L), which indicates the severity of the potential toxic impacts of CYP on fish in the wild. Rohu was observed to be vulnerable against CYP at an acute concentration. The results of the current study can be interpolated on other commercially valuable cyprinids as well. Therefore, the extensive use of CYP should be restricted, controlled, avoided and strictly monitored so as to ensure a thriving wild population of rohu, a commercially valuable major carp, as well as other aquatic organisms. Moreover, vitamin C can be included in fish feed to enhance their immunity and help them to cope with contaminants and stress under conditions of elevated ROS production.

## Data Availability

All data associated with this study are provided in this article and its Supplementary Materials.

## Supplementary Materials

The following supporting information can be downloaded at: https://www.mdpi.com/article/10.3390/toxics12090664/s1, Table S1: Formulation of basal diet for rohu, *Labeo rohita* (35% protein); Figure S1: Percent DNA in head (%) of comets in peripheral erythrocytes of rohu, *Labeo rohita*. Data (mean ± S.E.; N = 9) were analyzed using ANOVA followed by HSK Tukey test. Different superscripted symbols on the bars show significant difference at *p* < 0.05; Figure S2: Fluorescent photomicrograph (40×) of peripheral blood erythrocytes after 96 h of exposure of rohu to LC_50_ of CYP (comet assay; stain—acridine orange).
