# Supplementary material for: Ameliorative Role of Vitamin C against Cypermethrin Induced Oxidative Stress and DNA Damage in Labeo rohita (Hamilton, 1822) Using Single Cell Gel Electrophoresis
Source: Toxics. 2024 Sep 12;12(9):664. doi: 10.3390/toxics12090664 (PMC11435545; doi:10.3390/toxics12090664)
Supplement: Supplementary file 1 [file toxics-12-00664-s001.zip › toxics-3153466-supplementary.pdf]

Table S1. Formulation of basal diet for rohu, Labeo rohita (35% protein)

Ingredients	Amount (g kg-1)
Soybean meal	212
Sunflower meal	212
Fish meal	105
Gluten 30%	105
Canola meal	212
Rice polish	52
Dicalcium phosphate (DCP)	10
Carboxy methyl cellulose (CMC)	10
Vitamin premix*	20
Wheat bran	52
Vegetable oil	10

*(Vitamin premix contains vitamins, amino acid and minerals premix kg-1)

Vitamin AB.P 40,000,000 IU, Vitamin K3B.P 800 mg, Vitamin D3B.P 820,000 IU, Vitamin EB.P 6200 mg, Vitamin B3B.P 5100 mg, Vitamin B2B.P 2500 mg, Vitamin B12B.P 1000 mg, Vitamin PP B.P 10,500 mg, DL-Methionine B.P 50,500 mg, L. lysine B.P 10,500 mg, Manganese USP 30,000 mg 15,100 mg, Zinc USP 17,555 mg, Copper B.P 1000 mg, Choline chloride USP 125,500 mg, Cobalt B.P 50 mg, Iodine B.P 300 mg, Selenium B.P 80 mg.

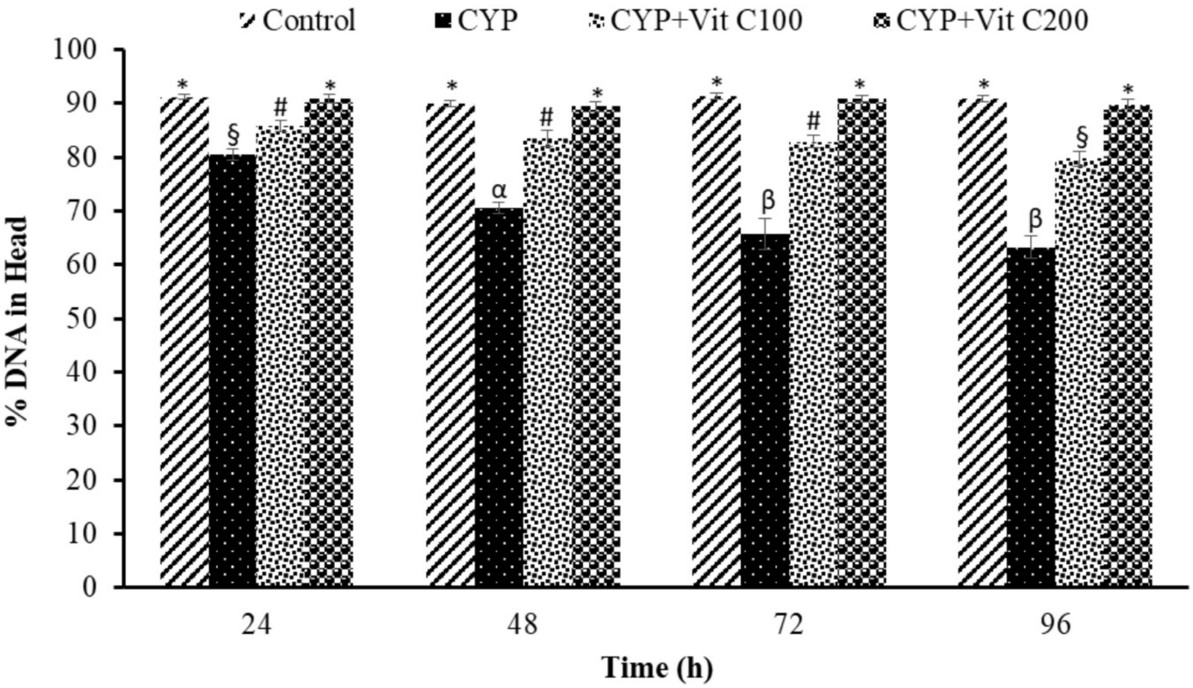

Figure S1. Percent DNA in head (%) of comets in peripheral erythrocytes of rohu, *Labeo rohita*. Data (mean ± S.E.; N = 9) was analyzed using ANOVA followed by HSK Tukey test. Different symbols on the bars show significant difference at $P < 0.05$ whereas the same symbol indicate no significant difference.

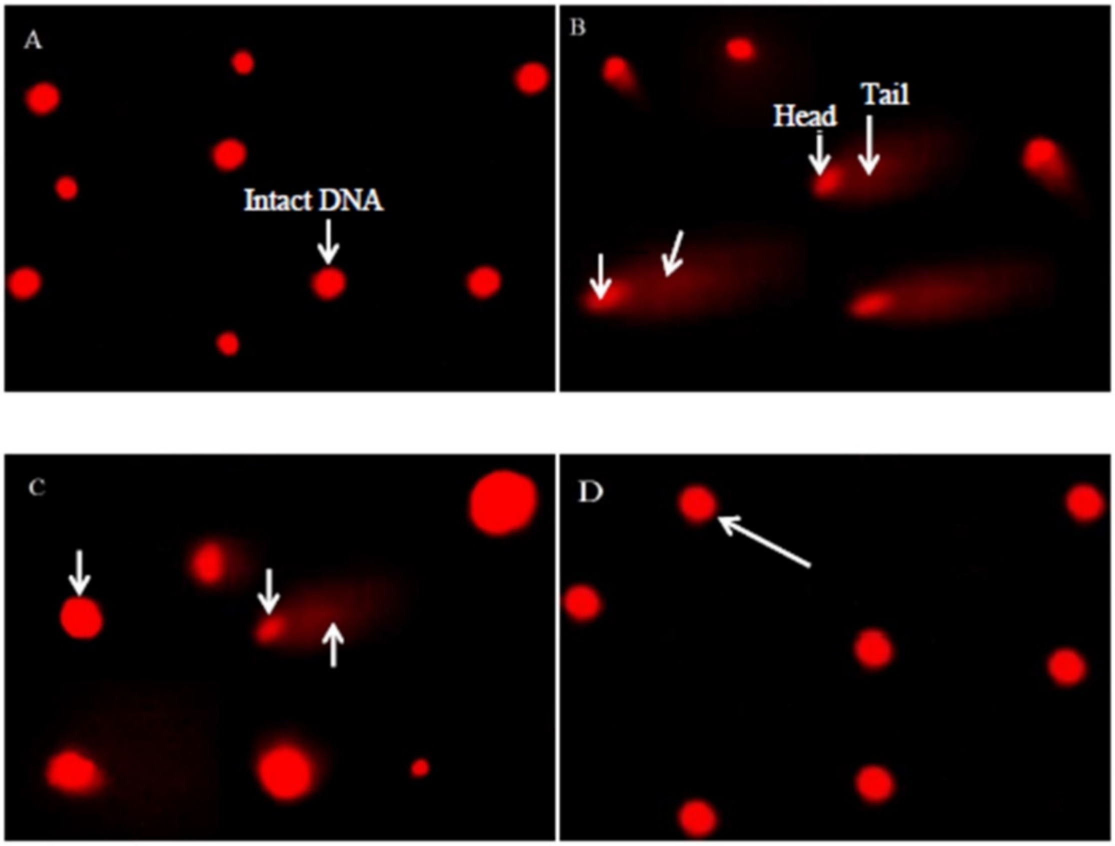

Figure S2. Fluorescent photomicrograph (40x) of peripheral blood erythrocytes after 96 hr of exposure of rohu to LC50 of CYP using comet assay (stain: Acridine orange). A) Intact DNA in Group 1st (Control). B) Comets with longer tail formed in Group 2nd (CYP alone). C) Comets with short tail and Intact DNA in Group 3rd (CYP+Vit C100 mg/kg diet). D) Intact DNA in rohu in Group 4th (CYP+Vit C200 mg/kg diet).
